# Zinc oxide nanoparticles conjugated with clinically-approved medicines as potential antibacterial molecules

**DOI:** 10.1186/s13568-021-01261-1

**Published:** 2021-07-10

**Authors:** Noor Akbar, Zara Aslam, Ruqaiyyah Siddiqui, Muhammad Raza Shah, Naveed Ahmed Khan

**Affiliations:** 1 College of Arts and Sciences, American University of Sharjah, University City, 26666 Sharjah, United Arab Emirates; 2 International Centre for Chemical and Biological Sciences, H.E.J. Research Institute of Chemistry, University of Karachi, Karachi, 75270 Pakistan; 3 Department of Clinical Sciences, College of Medicine, University of Sharjah, University City, 27272 Sharjah, United Arab Emirates

**Keywords:** Antibiotic resistance, Infectious diseases, ZnO-nanoparticles, Cytotoxicity

## Abstract

At present, antibiotic resistance is one of the most pressing issues in healthcare globally. The development of new medicine for clinical applications is significantly less than the emergence of multiple drug-resistant bacteria, thus modification of existing medicines is a useful avenue. Among several approaches, nanomedicine is considered of potential therapeutic value. Herein, we have synthesized Zinc oxide nanoparticles (ZnO-NPs) conjugated with clinically-approved drugs (Quercetin, Ceftriaxone, Ampicillin, Naringin and Amphotericin B) with the aim to evaluate their antibacterial activity against several Gram-positive (Methicillin resistant *Staphylococcus aureus, Streptococcus pneumoniae* and *Streptococcus pyogenes*) and Gram-negative (*Escherichia coli* *K1*, *Serratia marcescens* and *Pseudomonas aeruginosa*) bacteria. The nanoparticles and their drug conjugates were characterized using UV-visible spectrophotometry, dynamic light scattering, Fourier transform infrared spectroscopy and atomic force microscopy. Antibacterial activity was performed by dilution colony forming unit method and finally 3-(4,5-dimethylthiazole-2-yl)-2,5-diphenyltetrazolium bromide (MTT) assays were performed to determine their cytotoxic effects against human cell lines. ZnO-NPs revealed maxima surface plasmon resonance band at 374 and after conjugation with beta-cyclodextrin at 379 nm, polydispersity with size in range of 25–45 nm with pointed shaped morphology. When conjugated with ZnO-NPs, drug efficacy against MDR bacteria was enhanced significantly. In particular, Ceftriaxone- and Ampicillin-conjugated ZnO-NPs exhibited potent antibacterial effects. Conversely, ZnO-NPs and drugs conjugated NPs showed negligible cytotoxicity against human cell lines except Amphotericin B (57% host cell death) and Amphotericin B-conjugated with ZnO-NPs (37% host cell death). In conclusion, the results revealed that drugs loaded on ZnO-NPs offer a promising approach to combat increasingly resistant bacterial infections.

**Supplementary Information:**

The online version contains supplementary material available at 10.1186/s13568-021-01261-1.

## Key points

- Zinc oxide nanoparticles were conjugated successfully with several medicines.
- ZnO-NPs-conjugated drugs exhibited potent cidal effects against MDR bacteria.
- Human cells were not affected by ZnO-NPs and drugs conjugated NPs.
- Nanomedicine hold promise in the treatment of infections caused by MDR bacteria.

## Introduction

The emergence and spread of drug-resistant microbes is far more rapid than the introduction of new medicines into clinical practice (Ling et al. 2015). In recent decades, the list of multiple drug-resistant (MDR) bacteria have grown at an alarming rate associated with a growing list of infections, such as pneumonia, tuberculosis, diarrhea, septicemia, gonorrhoea, meningitis etc. (Khameneh et al. 2016). In part, this is due to significant genetic flexibility observed in bacteria that enable them to respond to wide-ranging ecological threats, especially wider use of antibacterial compounds in animal feed and to treat routine infections without prescription (Blair et al. 2015; Munita and Arias 2016). The discovery and introduction of novel antibacterial agents in the clinical practice is a lengthy and expensive process. An alternative approach is to modify existing drugs to enhance their efficacy. In this regard, nanotechnology-based nanomedicine is a promising approach in eradicating bacterial infections (Mukheem et al. 2018; Ssekatawa et al. 2020). Nanoparticles (NPs) have demonstrated antibacterial activity against a variety of MDR bacteria by increasing the surface area, enhancing release of drug, reducing the dose required, and improving solubility and bioavailability of drugs (Wang et al. 2017; Anwar et al. 2018). Several nanoparticles have been tested as delivery vehicles comprising of dendrimers, liposomes, metallic nanoparticles, and polymeric nanoparticles. Metal nanoparticles have intrinsic physicochemical and optical properties, which enable them to be used for various applications (Souza et al. 2020). Notably, Zinc oxide NPs (ZnO-NPs) have been used on nano as well as microscale in different formulations and considered safe against human cells (Sirelkhatim et al. 2015). ZnO-NPs possess unique semiconducting properties, which is the main cause of their utilization in electronics and biomedical field. ZnO-NPs have inherent antibacterial potential and has been declared as biocompatible antimicrobials by FDA (Souza et al. 2020). Moreover, ZnO-NPs are cost-effective and their ease of functionalization is highly feasible for the development of antibacterial formulations (Jones et al. 2008). Beta-cyclodextrins (BCDs) are biocompatible cyclic oligosaccharides having R-(1,4)-linked-D-glucopyranose units arranged in ring form. BCDs have unique ability to form inclusion complexes with organic molecules via host–guest interactions and hence widely utilized in drug delivery applications (suitable drug carriers) for the enhancement of solubility and bioavailability of poor water-soluble drugs (Davis and Brewster 2004; Ren et al. 2017).

To determine whether ZnO-NPs exhibit antibacterial effects against MDR bacteria and whether conjugation of ZnO-NPs can enhance efficacy of drugs, several drugs were used. These include Quercetin (molar mass 302.2 g mol^−1^), a plant-based flavonoid with antibacterial activity. It showed bacteriostatic effects against *P. aeruginosa, E. coli, S. enterica* and *S. aureus* (Wang et al. 2018). Ceftriaxone (molar mass 554.6 g mol^−1^) belong to cephalosporin family with potent antibacterial properties (Ebrahimi et al. 2020). It is used to treat a wide-range of bacterial infections including; meningitis, intra-abdominal, middle ear, joint, bone and skin infections respectively (Kumar et al. 2016). Ceftriaxone kill bacteria by inhibiting the cross-linking of peptidoglycan and ultimately stop cell wall synthesis (Hathout et al. 2020). Ampicillin (molar mass 349.41 g mol^−1^), a beta lactam antibacterial drug is used to treat infections caused by Gram-positive and Gram-negative bacteria. Ampicillin inhibit the transpeptidases needed in cell wall synthesis followed by bacterial cell lysis (Tipper 1985; Kaushik et al. 2014). Naringin (580.541 g mol^−1^) is a flavonoid used to target the quorum sensing mechanism and inhibit *Aeromonas hydrophila* (Srinivasan et al. 2020). Amphotericin B (molar mass 924.079 g mol^−1^) is used as effective antifungal that inhibits the formation of cell walls of young fungal cells (Grela et al. 2019).

In the present study, ZnO-NPs were synthesized by direct precipitation method and after successful formation the NPs were loaded with beta-cyclodextrin and finally conjugated with different drugs to form ZnO-CD-Drug complex. The NPs itself and their conjugates were characterized by several characterization techniques such as zeta sizer and zeta potential analysis, UV-visible, FTIR and AFM. Furthermore, the NPs and drug-NPs conjugates were evaluated for their antibacterial activities against Gram-positive and Gram-negative MDR bacteria. Finally, their cytotoxicity/viability were assessed using human cell lines. The conjugation of drugs with ZnO-NPs exhibited remarkable antibacterial activities with minimal human cells cytotoxicity. These are remarkable findings and should pave the way in the formulations of new antibacterials.

## Materials and methods

Zinc acetate dihydrate*,* sodium hydroxide, beta-cyclodextrin (BCD), quercetin (QT) and naringin (NAR) were purchased from Sigma-Aldrich. Ceftriaxone (CFT), ampicillin (AMP) and amphotericin (AMB) were acquired from Merck. HPLC-grade methanol and acetone were used. Deionized water was used for making solutions. All chemicals were utilized without pre-treatment or further purification.

### Preparation of ZnO nanoparticles (ZnO NPs)

Zinc oxide nanoparticles were synthesized by utilizing zinc acetate dihydrate and NaOH as precursors using direct precipitation method as previously described (Raoufi 2013; Ghorbani et al. 2015). Aqueous solutions of zinc acetate dehydrate (0.1 M), and sodium hydroxide (0.2 M) were prepared. Both solutions were added drop wise into a beaker at room temperature with continuous stirring at 600 rpm for 2 h. The resulting white precipitation was separated by centrifugation (Minispin Plus, Eppendorf^®^) for 15 min at 10,000×*g* and subsequently washed three times with deionized water followed by acetone washing. The precipitates were dried in oven at 120 °C for 6 h and then calcinated at 300 °C in air atmosphere to obtain stable ZnO-NPs.

### Preparation of beta-cyclodextrin capped ZnO nanoparticles (BCD-ZnO NPs)

For the preparation of BCD-ZnO NPs complex, 30 mg of ZnO nanoparticles were dispersed in 10 mL of deionized water and sonicated for 15 min. Next, 30 mg beta-cyclodextrin was dissolved in 10 mL of deionized water and stirred at 600 rpm for 10 min to obtain a transparent solution. Both solutions were mixed under continuous stirring at 600 rpm. After six hours, beta-cyclodextrin capped ZnO nanoparticles (ZnO-BCD NPs) were separated by centrifugation (Minispin Plus, Eppendorf^®^) for 15 min at 8000×*g* and washed three times with deionized water. The supernatant was freeze-dried, and the residue weighed, which indicated that beta-cyclodextrin capped ZnO nanoparticles contained about 30.5% by weight of beta-cyclodextrin. Beta-cyclodextrin capped ZnO nanoparticles (BCD-ZnO NPs) were dried in oven below 80 °C.

### Preparation of drug-loaded beta-cyclodextrin capped ZnO nanoparticles (ZnO-BCD NPs)

Five  mL solution of five drugs (Quercetin, naringin, ceftriaxone, ampicillin and amphotericin B) was prepared in methanol having concentration 1 mg/mL.5 mg ZnO-BCD NPs were added in each five-drug solutions separately upon continuous stirring at 600 rpm. After 24 h, the suspension was centrifuged (Minispin Plus, Eppendorf^®^) at 8000×*g* for 15 min to obtain drug (Quercetin, naringin, ceftriaxone, ampicillin and amphotericin B) loaded beta-cyclodextrin capped ZnO nanoparticles. Drug loaded nanoparticles were dried at room temperature and suspended in deionized water for further characterization.

### Characterization of ZnO NPs, BCD-ZnO NPs and drug loaded BCD-ZnO NPs

ZnO nanoparticles (ZnO NPs), beta-cyclodextrin capped ZnO nanoparticles (BCD-ZnO NPs) and drug loaded BCD-ZnO NPs were characterized with UV-visible spectroscopy, Fourier transform infrared spectroscopy (FTIR), Atomic Force Microscopy, zeta sizer and zeta potential analysis. The particle size, size distribution and zeta potential of ZnO nanoparticles (ZnO NPs), beta-cyclodextrin capped ZnO nanoparticles (BCD-ZnO NPs) and drug loaded BCD-ZnO NPs was determined by dynamic light scattering (DLS) (Malvern, Zetasizer Nano ZSP) as described previously (Yusof et al. 2019)*.* Sample was prepared in deionized water, passed through syringe filter (0.45 µm) and analyzed for size, poly dispersity index (PDI) and zeta potential determination. FTIR spectra of ZnO NPs, BCD-ZnO NPs and drug loaded BCD-ZnO NPs were recorded by Shimadzu IR-470 spectrometer (Shimadzu, Kyoto) in the range of 4000–400 cm^−1^ by using KBr disk method as described earlier (Yusof et al. 2019). Sample was grinded with KBr 1% (w/w) and pressed by applying mechanical pressure to obtain KBr disk.

### Drug encapsulation efficiency of ZnO-BCD NPs

Five drugs (Quercetin, naringin, ceftriaxone, ampicillin and amphotericin B) were separately loaded on to ZnO-BCD NPs (Additional file 1: Figure S1). Approximately 5 mg of ZnO-BCD NPs were suspended in 5 mL of methanol containing each drug (1 mg/mL). The resulting suspension was stirred (600 rpm) at room temperature for 24 h. Drug loaded ZnO-BCD NPs were collected by centrifugation and the supernatant was analyzed by using UV-visible spectrophotometer (Shimadzu, UV-1800) for the evaluation of amount of un-encapsulated drug. The encapsulation efficiency of drug loaded ZnO-BCD NPs was calculated by the given formula.

$${\text{Drug encapsulation efficiency}} = \frac{{{\text{Total amount of drug}} - {\text{Free drug in the supernatant~}}}}{{{\text{Total amount of drug}}}} \times 100$$

### Bacterial cultures

Bacterial cultures used in the study are shown in (Table 1). The bacteria include methicillin resistant *Staphylococcus aureus* (MRSA), *Streptococcus pyogenes* and *S. penumoniae* (Gram-positive), *Escherichia coli* K1, *Pseudomonas aeruginosa* and *Serratia marcescens* (Gram-negative). All the bacterial isolated were cultured and revived at 37 °C for overnight prior the experiments.

**Table 1:** Bacterial isolates used in this study

Bacterial isolate	Strain
Methicillin resistant *Staphylococcus aureus*	MTCC 381123 (clinical isolate)
*Streptococcus pyogenes*	ATCC 49399 (clinical isolate)
*Streptococcus pneumoniae*	ATCC 13883 (clinical isolate)
*Pseudomonas aeruginosa*	ATCC 10145 (clinical isolate)
*Escherichia coli* K1	MTCC 710859 (clinical isolate)
*Serratia marcescens*	MTCC 13880 (clinical isolate)

### Antibacterial assays

Antibacterial assays were performed to determine the bactericidal activities of the drugs and drug conjugated nanoparticles (Table 2) against a panel of Gram-positive and Gram-negative bacteria as previously described (Akbar et al. 2018, 2020). Briefly, 1  ×  10^6^ bacterial cells were incubated with 100 µg/mL of the drugs and drug conjugated NPs for 2 h at 37 °C. Next, the cultures were serially diluted ten-fold and different dilutions (i.e., 10^–3^–10^–6^) were plated on freshly prepared nutrient agar plates. The plates were incubated for overnight at 37 °C and the viable bacterial colonies were enumerated. For negative control, bacteria alone were incubated in saline whereas, bacteria incubated with 100 µg/mL of Gentamicin was taken as positive control.

**Table 2:** Concentration of drugs and drug loaded ZnO-NPs used in this study

S. no.	Sample code of pure drug/formulation	Pure drug/formulation	Concentration µg/mL
1	QTG	Quercetin	100
2	CEFT	Ceftriaxone	100
3	NAR	Naringin	100
4	AMB	Amphotericin B	100
5	AMPI	Ampicillin	100
6	ZnO-CD	ZnO-cyclodextrin conjugate nanoparticles	100
7	Zn–O	ZnO-nanoparticle	100
8	CD	Beta-cyclodextrin	100
9	ZnO-CD-AMPI	ZnO-cyclodextrin-Ampicillin conjugates	100
10	ZnO-CD-CFT	ZnO-cyclodextrin-CFT conjugates	100
11	ZnO-CD-NAR	ZnO-cyclodextrin-Naringin conjugates	100
12	ZnO-CD-AMB	ZnO-cyclodextrin-Amphotericin B conjugates	100
13	ZnO-CD-QTG	ZnO-cyclodextrin-Quercetin conjugates	100

Next, minimum inhibitory concentration (MIC_50_) of ZnO and drugs loaded NPs was determined using broth micro-dilution assays as previously described (Emami-Karvani and Chehrazi 2011; Tamboli and Lee 2013). Briefly, 1  ×  10^5^ bacteria were incubated with ZnO-NP and drugs loaded ZnO-NPs at various concentrations ranging from 6.25 to 800 µg/mL (two-fold diluted in Muller Hinton Broth) at 37 °C for overnight. Bacteria alone in MHB was used as positive control while MHB alone was used as negative control. After this incubation, MIC_50_ was calculted by measuring the optical density at 600 nm using Tecan plate reader. The lowest concentration of NPs and drug-NPs conjugates that inhibited 50% of growth of test bacteria was measured as MIC_50_.

### Cell viability assays

Cell viability assays were performed to determine the cytotoxic properties of the drugs and drug-loaded NPs against human cell lines using 3-(4,5-dimethylthiazol-2-yl)-2,5-diphenyltetrazolium bromide or MTT assays as described earlier (Anitha et al. 2018; Akbar et al. 2019). Briefly, cell lines were grown in a 96 well plates for overnight at 37 °C in the presence of 5% CO_2_ in a humidified incubator. Upon 80–90% confluency, the cell monolayer was challenged with a range of concentration (12.5, 25, 50, 100, 200 and 400 µg/mL) of the drugs and drug conjugates at 37 °C in the presence of 95% humidity and 5% CO_2_ for 24 h. After this incubation, 10 µL of freshly made MTT solution was added to each well and further incubated for 4 h. Next, 100 µL of DMSO was added to each well to dissolve the formazan crystals formed by the live cells. For negative control, DMSO was added to the well with no drug. Finally, the absorbance was recorded at 540 nm and values were interpreted to calculate the percentage inhibition/viability by following equation:

$$Mean~OD~of~test~sample/MeanOD~of~negative~control~ \times 100$$

### Statistical analysis

For statistical analysis, GraphPad Prism version 8.0.2 (GraphPad Software, San Diego, CA, USA) was used. The data are expressed as the mean  ±  standard error of several independent experiments performed in duplicates. P values were calculated using Student’s T test (two-tailed distribution) where P value  ≤  0.05 was considered as statistically significant.

## Result

### UV–Visible absorption spectra of ZnO NPs and BCD-ZnO NPs and drug-BCD-ZnO NPs

**Fig. 1:**
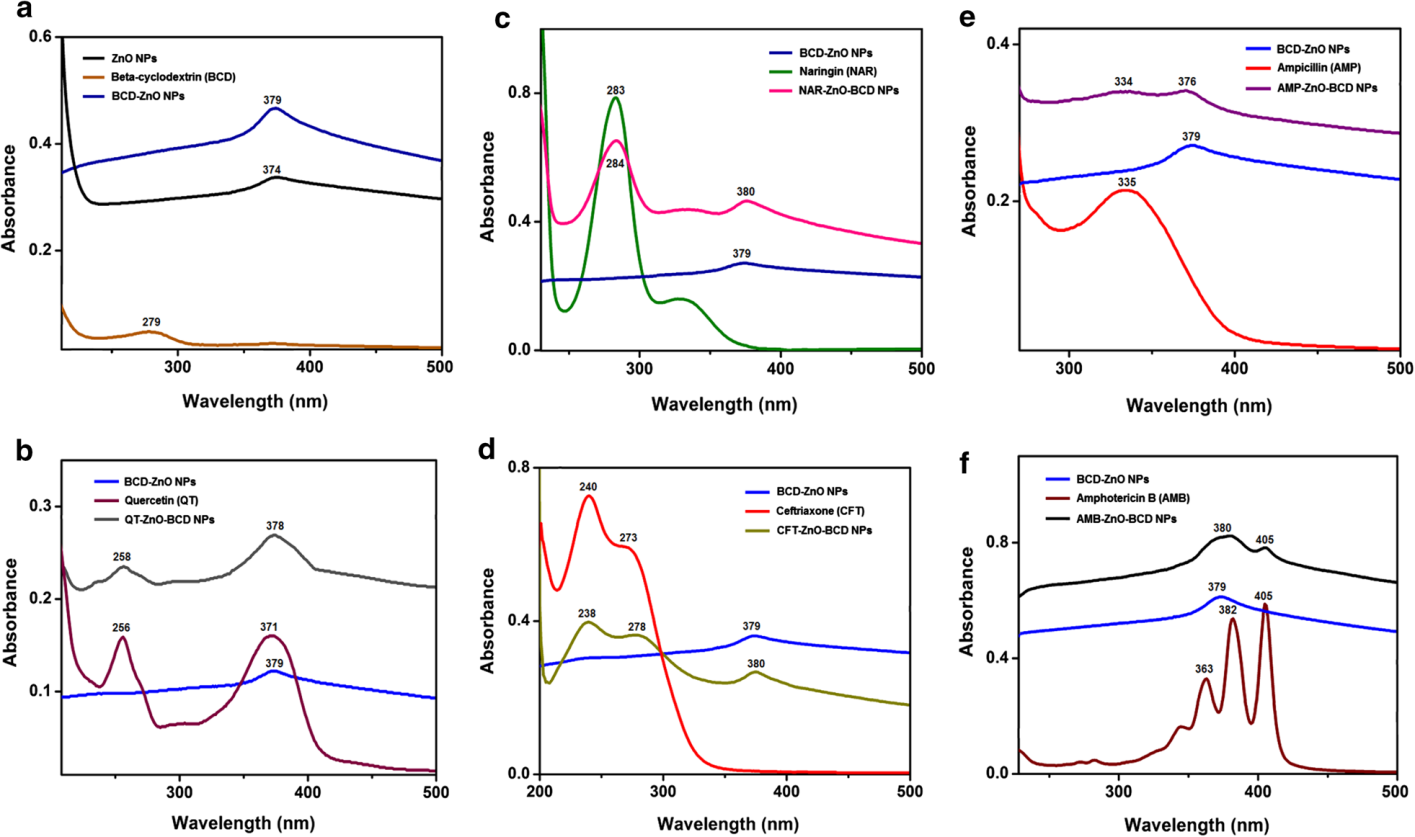
Representing the UV-visible absorption spectra of nanoparticle, reducing/capping agent and different drugs conjugates. **a** Represents UV spectra of ZnO NPs, beta-cyclodextrin (BCD) and BCD-ZnO NPs, **b** showing spectra of BCD-ZnO NPs, quercetin and QT-BCD-ZnO NPs, **c** absorption spectra of BCD-ZnO NPs, naringin and NAR-BCD-ZnO NPs, **d** absorption spectra of BCD-ZnO NPs, ceftriaxone and CFT-BCD-ZnO NPs, **e** absorption spectra of BCD-ZnO NPs, ampicillin and AMP-BCD-ZnO NPs and **f** illustrating absorption spectra of BCD-ZnO NPs, amphotericin B and AMB-BCD-ZnO NPs

UV-visible spectroscopic analysis was performed to evaluate the changes occurred in the UV-visible absorption of ZnO NPs after capping of beta-cyclodextrin over their surface (Fig. 1). UV-visible absorption spectrum of ZnO nanoparticles (black), beta-cyclodextrin (orange) and beta-cyclodextrin capped ZnO NPs (ZnO-BCD NPs, blue) is shown in (Fig. 1a). The absorption peak at 374 nm is the characteristic peak for hexagonal wurtzite ZnO. Beta-cyclodextrin exhibited peak at 279 nm. After the capping of ZnO NPs with beta-cyclodextrin, the UV-visible absorbance of ZnO-BCD NPs red shifted towards 379 nm, which indicated the interaction of beta-cyclodextrin with ZnO NPs surface causes defects in the crystal lattice of ZnO-NPs due to the presence of beta-cyclodextrin atoms in the lattice. Similarly, UV–Vis spectra for all the five drugs conjugated with ZnO-BCD NPs are shown in (Fig. 1b–f) confirming the successful interaction of drugs and ZnO-BCD NPs (Kadam et al. 2020). Quercetin exhibited two major peaks around 256 nm and 371 nm which are associated with the cinnamoyl and benzoyl systems present in the basic structure of QT (Catauro et al. 2015). After the loading of quercetin (Fig. 1b), QT-ZnO-BCD NPs showed peaks at 258 nm and 378 nm, the peak at 378 nm is broadened which might be due to the merging of peaks of benzoyl system of QT and BCD-ZnO NPs. Naringin absorption peak appeared at 283 nm corresponding to the cinnamoyl system (Diaz-Uribe et al. 2016; Fig. 1c) while after the formation of NAR-ZnO-BCD NPs, two peaks appeared at 284 nm and 380 nm which are the characteristics peaks of naringin and BCD-ZnO NPs. Ceftriaxone absorption peak appeared at 240 nm (Ethiraj et al. 2014, Fig. 1d) and CFT-ZnO-BCD NPs showed peaks at 238 and 380 nm. Absorption peak of ampicillin appeared at 335 nm (Ambekar et al. 2015) while AMP-ZnO-BCD NPs showed two peaks at 334 nm and 376 nm (Fig. 1e). Amphotericin B showed three characteristic peaks at 363 nm, 382 nm and 405 nm (Vandermeulen et al. 2006) while after the formation of AMB-ZnO-BCD NPs, two peaks appeared at 380 nm and 405 nm (Fig. 1f). Calibration curve are shown for quercetin (Additional file 1: Figure S2), naringin (Additional file 1: Figure S3), ceftriaxone (Additional file 1: Figure S4), ampicillin (Additional file 1: Figure S5), and amphotericin B (Additional file 1: Figure S6).

### Size distribution and zeta potential analysis of ZnO NPs and BCD-ZnO NPs

**Fig. 2:**
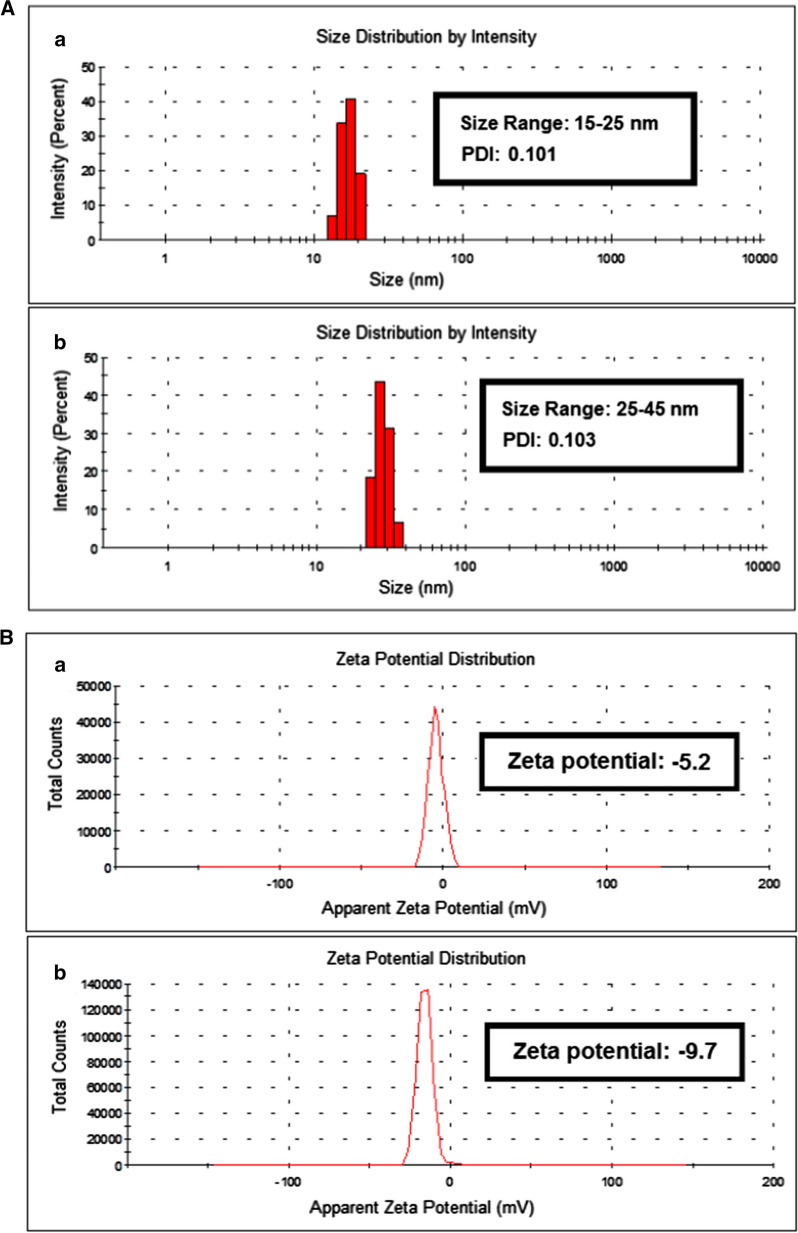
Showing the average particle size distribution histogram and zeta potential of ZnO nanoparticle and the reducing/capping agent **A** depicts average particle size distribution **a** ZnO nanoparticles (ZnO NPs) and **b** beta-cyclodextrin capped ZnO NPs (ZnO-BCD NPs) and **B** showing zeta potential graph of **a** ZnO nanoparticles (ZnO NPs) and **b** beta-cyclodextrin capped ZnO NPs (ZnO-BCD NPs)

**Fig. 3:**
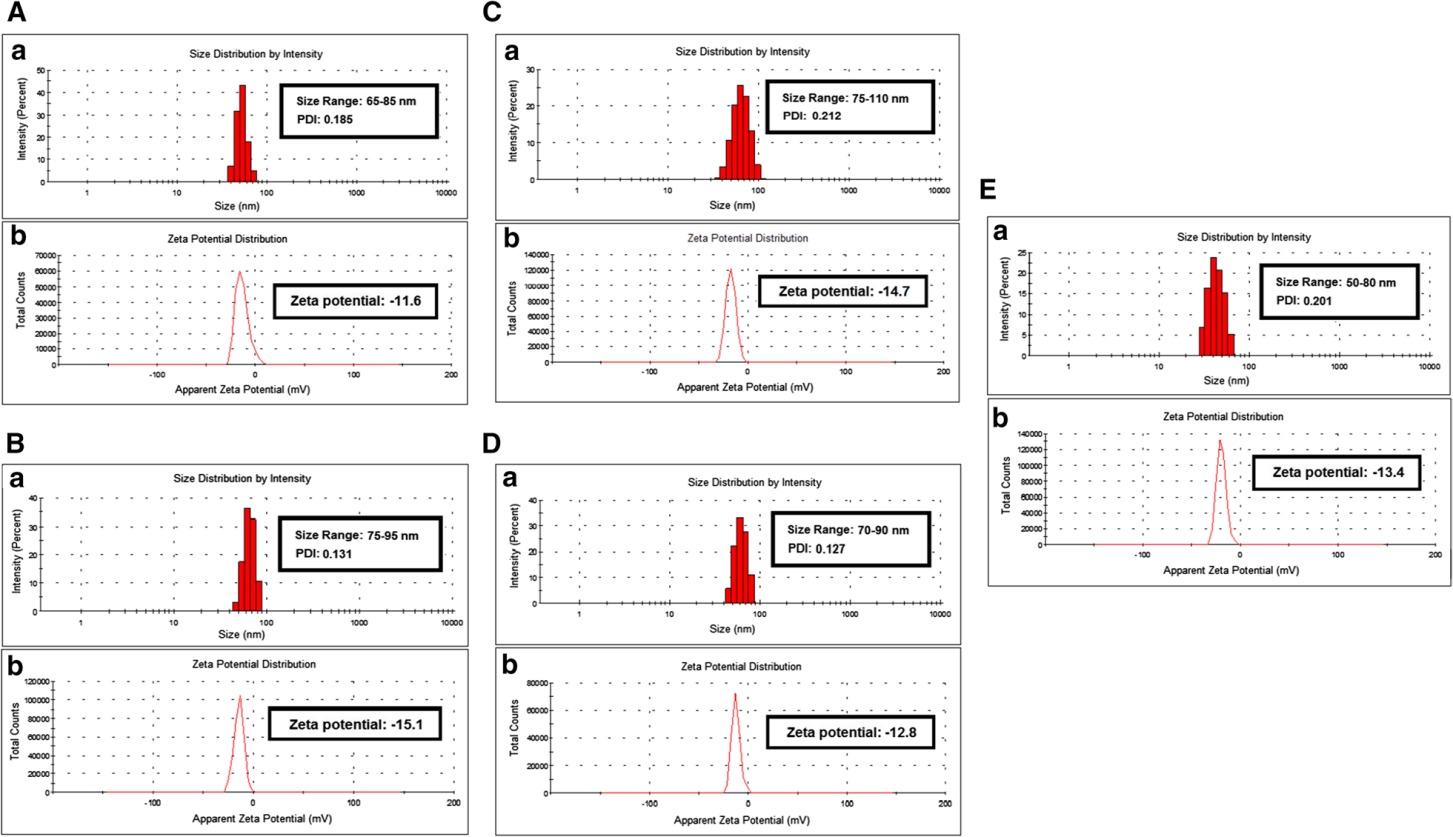
Illustrating the average particle size distribution histogram and zeta potential of ZnO nanoparticle, the reducing/capping agent and various drugs used in the study where **A** showing particle size (**a**) and zeta potential graph (**b**) of quercetin loaded beta-cyclodextrin capped ZnO NPs (QT-ZnO-BCD NPs), **B** showing particle size distribution histogram (**a**) and zeta potential graph (**b**) of naringin loaded beta-cyclodextrin capped ZnO NPs (NAR-ZnO-BCD NPs), **C** display average particle size distribution histogram (**a**) and zeta potential graph (**b**) of ceftriaxone loaded beta-cyclodextrin capped ZnO NPs (CFT-ZnO-BCD NPs), **D** display particle size distribution histogram (**a**) and zeta potential graph (**b**) of ampicillin loaded beta-cyclodextrin capped ZnO nanoparticles (AMP-ZnO-BCD NPs) and **E** showing particle size distribution histogram (**a**) and zeta potential graph (**b**) of amphotericin B loaded beta-cyclodextrin capped ZnO nanoparticles (AMB-ZnO-BCD NPs)

Particle size distribution of nanocarriers plays a major role for the design of drug delivery systems. It has been reported that particle size affects the pattern of drug release (Liu et al. 2015). Smaller particles have larger surface area which leads to optimum loading of drug as compared to particles having larger size distribution. Zeta sizer analysis was carried out for the evaluation of average particle size of ZnO nanoparticles (ZnO NPs) and beta-cyclodextrin capped ZnO NPs (BCD-ZnO NPs). The results are presented in Fig. 2. The average particle size of ZnO NPs was found in the range of 15–25 nm (Fig. 2) having polydispersity index (PDI) 0.101. This result showed that the ZnO nanoparticles are highly monodisperse and have uniform size range (Kavitha et al. 2017). After the capping of beta-cyclodextrin over the surface of ZnO nanoparticles the average particle size distribution of BCD-ZnO NPs increased and was found to be in the range of 25–45 nm with PDI 0.103 (Fig. 2). The increased polydispersity of nanoparticles lead to increase in size distribution of nanoparticles due to the surface interaction of beta-cyclodextrin with ZnO NPs. Surface charges present on the nanoparticles are important as they determine the interaction of nanocarriers with the biological environment as well as their behavior with bioactive compounds. Zeta potential analysis was carried out for the evaluation of surface charges and stability of ZnO nanoparticles (ZnO-NPs) and beta-cyclodextrin capped ZnO NPs (BCD-ZnO NPs). Zeta potential graph of ZnO-NPs and BCD-ZnO NPs are shown in (Fig. 3). Zeta potential of ZnO nanoparticles was found to be − 5.2 and BCD-ZnO NPs was − 9.7. This increase in zeta potential indicated that the interaction of beta-cyclodextrin with ZnO nanoparticles causes enhancement in the surface charges and lead to greater stability of BCD-ZnO NPs. Higher surface charges on nanoparticles prevents aggregation of formulation due to greater repulsion among the nanoparticles. The result of dynamic light scattering (DLS) analysis of ZnO NPs and BCD-ZnO NPs are summarized in Table 3.

**Table 3:** Size, size distribution, zeta potential of ZnO NPs and BCD-ZnO NPs and encapsulation efficiency of drug (quercetin, naringin, ceftriaxone, ampicillin and amphotericin B) loaded BCD-ZnO NPs

S. no.	Nanoparticles (NPs)	Size range (nm)	Polydispersity Index (PDI)	Zeta potential (mV)	Drug encapsulation efficiency (EE%)
1	ZnO NPs	15–25	0.101	− 5.2	–
2	BCD-ZnO NPs	25–45	0.103	− 9.7	–
1	QT-ZnO-BCD NPs	65–85	0.185	− 11.6	52.5
2	NAR-ZnO-BCD NPs	75–95	0.131	− 15.1	44.5
3	CFT-ZnO-BCD NPs	75–110	0.212	− 14.7	47.5
4	AMP-ZnO-BCD NPs	70–90	0.127	− 12.8	40.0
5	AMB-ZnO-BCD NPs	50–80	0.201	− 13.4	49.5

Five drugs (Quercetin, naringin, ceftriaxone, ampicillin and amphotericin B) were separately loaded over the surface of ZnO-NPs by following the procedure explained in the experimental section (Additional file 1: Figure S1). After the drug loading, zeta sizer and zeta potential analysis was carried out to observe the changes occurred in particle size and surface charges on drug loaded BCD-ZnO NPs nanoparticles. The results are summarized in the Table 3. As expected, the size and zeta potential of the nanoparticles after the loading of drug increases as shown in Table 3 (Kaur et al. 2019). Surface charges tends to increase due to the interaction of drug molecules with the surface of BCD-ZnO NPs.

### FTIR analysis

**Fig. 4:**
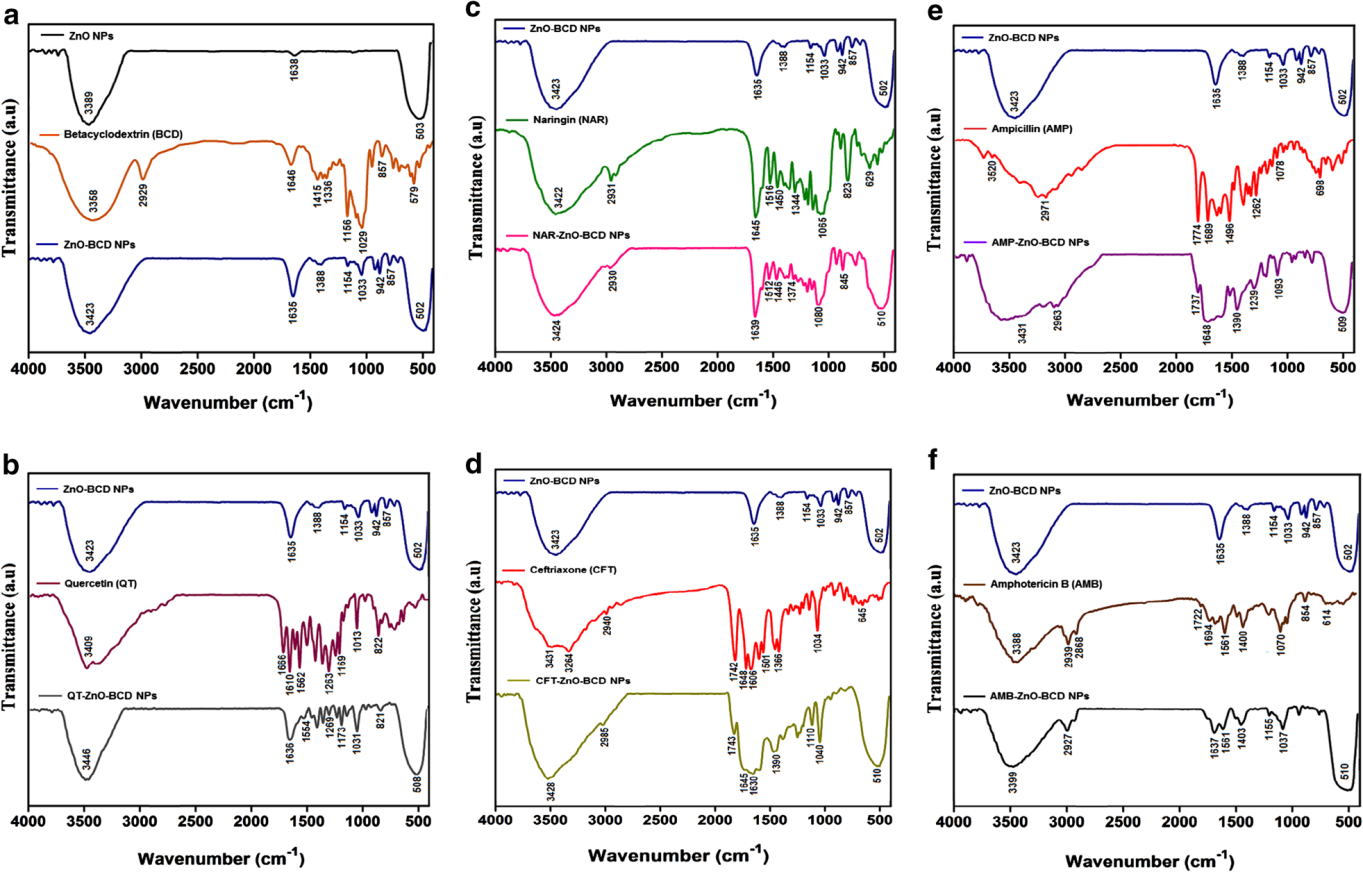
**a** FT-IR spectra of ZnO-NPs alone is compared with BCD and BCD-ZnO-NPs complex, **b** relative FT-IR analysis of (ZnO-BCD NPs), quercetin (QT) and (QT-ZnO-BCD NPs), **c** comparative FTIR analysis of (ZnO-BCD NPs), naringin (NAR) and (NAR-ZnO-BCD NPs), **d** FTIR analysis of (ZnO-BCD NPs), ceftriaxone (CFT) and (CFT-ZnO-BCD NPs), **e** FTIR analysis of (ZnO-BCD NPs), ampicillin (AMP) and (AMP-ZnO-BCD NPs) and **f** FTIR analysis of (ZnO-BCD NPs), amphotericin B (AMB) and (AMB-ZnO-BCD NPs). All the spectra were acquired by employing IR-470 spectrometer (Shimadzu, Kyoto, Japan)

Comparative FTIR spectra of ZnO nanoparticles (ZnO NPs), beta-cyclodextrin (BCD) and beta-cyclodextrin capped ZnO nanoparticles (ZnO-BCD NPs) is shown in (Fig. 4a–f). FTIR of ZnO nanoparticles (black) showed broad peak in the region of 3400–3300 cm^−1^ which corresponds to the O–H bond while insignificant peak at 1638 cm^−1^ might be due to the acetate group, precursor used for the synthesis of ZnO nanoparticles (Nagaraju et al. 2017; Fig. 4a). Significant peak in the region of 550–400 cm^−1^ is the characteristic peak at of Zn–O bond pessent in the ZnO nanoparticles (Nagaraju et al. 2017). Beta-cyclodextrin FTIR spectrum (orange) showed broad peak in the region of 3500–3300 cm^−1^ which is the characteristic peak of O–H group while peak at 2929 cm^−1^ correspond to the stretching frequency of C–H bond (Rachmawati et al. 2013). IR absorption peaks at 1646 cm^−1^, 1156 cm^−1^, 976 cm^−1^ and 853 cm^−1^ are attributed to the H–O–H, C–O, C–O–C (glucose units) and C–O–C bond of rings of beta-cyclodextrin, respectively (Reddy et al. 2020). FTIR spectrum (blue) of beta-cyclodextrin capped ZnO nanoparticles (ZnO-BCD NPs) showed broad peak in the region of 3500–3200 cm^−1^ which is the characteristic peak of O–H groups while a significant characteristic absorption peak at 1635 cm^−1^ is appearing due to H–O–H bond of beta-cyclodextrin. Peaks at 1154 cm^−1^, 942 cm^−1^ and 857 cm^−1^ are appearing due to the C–O, C–O–C (glucose units) and C–O–C bond of beta-cyclodextrin attached over the surface of ZnO-BCD NPs. Most significant peak in the region of 550–400 cm^−1^ corresponds to the Zn–O bond (Sawant and Bamane 2018). Appearance and slight shift of beta-cyclodextrin peaks in the FTIR speactrum of ZnO-BCD NPs, confirms the successful capping of ZnO with beta-cyclodextrin (Fig. 4a). After loading of all five drugs (Quercetin, naringin, ceftriaxone, ampicillin and amphotericin B) over beta-cyclodextrin capped ZnO nanoparticles (ZnO-BCD NPs), FTIR analysis was performed to observe possible interactions of functional groups of each drug with ZnO-BCD NPs. The results are shown in (Figs. 2f–4b). The FTIR spectrum of quercetin exhibited broad peak at 3409 cm^−1^ due to the phenolic hydroxyl groups (-OH) while band at 1666 cm^−1^ is due to the carbonyl (C = O) stretching frequency (Catauro et al. 2015; Fig. 4b). Absorption peaks at 1610 cm^−1^ and 1562 cm^−1^ are the characteristic C = C aromatic stretch bands. Peaks at 1263 cm^−1^ and 1013 cm^−1^ are appearing due to the stretching vibrations of C–O–C and C = C–O bonds, respectively (Catauro et al. 2015). The FTIR spectrum of QT-ZnO-BCD NPs showed peak in the region of 3500–3300 cm^−1^ which is due to the -OH group (Sathishkumar et al. 2021) while broad peak at 1636 cm^−1^ indicated the merging of peaks of quercetin C = C aromatic bonds and beta-cyclodextrin H–O–H bending vibrations. Suppression of peaks was observed at 1554 cm^−1^, 1269 cm^−1^ and 1031 cm^−1^ which are attributed to C = C, C–O–C and C = C–O bonds, respectively. Major absorption peak in the region of 550–400 cm^−1^ is attributed to the Zn–O bond (Sathishkumar et al. 2021). The peak shifting and suppression indicated the interaction of functional groups of quercetin with ZnO-BCD NPs and formation of QT-ZnO-BCD NPs (Fig. 4b).

After loading of Naringin over the surface of ZnO-BCD NPs, the FTIR spectra of NAR-ZnO-BCD NPs showed broad peak in the region of 3500–3300 cm^−1^ corresponding to the -OH group while peak at 2930 cm^−1^ is due to the stretching vibration of C–H bond (Yang et al. 2020) (Fig. 4c). Shifting of peaks was observed at 1639 cm^−1^ and 1446 cm^−1^ which are attributed to the stretching frequencies of C = O and –C = C– (aromatic) bond. Peak at 1080 cm^−1^ is the stretching vibration of –C–O– (polyols) groups of Naringin in NAR-ZnO-BCD NPs (Yang et al. 2020). The peak shifting observed in the absorption frequencies of C = O and –C–O– bond indicated the interaction of these functional groups with the surface of ZnO-BCD NPs (Fig. 4c).

The characteristic peaks of ceftriaxone appeared at 3431 cm^−1^ and 3264 cm^−1^ that corresponds to the asymmetric and symmetric stretching frequency of N–H group while absorption band at 2940 cm^−1^ is due to the stretching vibration of C–H group (Gunasekaran and Charles 2008; Fig. 4d). The stretching vibrations at 1742 cm^−1^, 1648 cm^−1^ and 1606 cm^−1^ corresponds to carbonyl of β-lactam (C = O), carbonyl of amide (C = O) and C = N bond of oxime, respectively (Gunasekaran and Charles 2008). The peak at 1034 cm^−1^ is due to the stretching vibration of C–O group. The broad peak for CFT-ZnO-BCD NPs in the region of 3500–3300 cm^−1^ and 2985 cm^−1^ shows O–H and C–H group, correspondingly (Mushtaq et al. 2017). Suppression of peak at 1743 cm^−1^ indicated the interaction of carbonyl (C = O) of β-lactam with the surface of BCD-ZnO NPs. Presence of peak at1645-1608 cm^−1^ related to vibrational frequencies of carbonyl of amide (C = O), C = N bond of oxime and H–O–H of BCD, respectively (Mushtaq et al. 2017). Peak 550–400 cm^−1^ is the characteristic peak of Zn–O bond. Suppression and shifting of peaks of ceftriaxone confirms the successful loading of drug over the surface of BCD-ZnO NPs (Fig. 4d).

FTIR of ampicillin showed broad peak in the region of 3500–3200 cm^−1^ which is due to the hydroxyl group, also the shoulder speaks at 3347 cm^−1^ and 3358 cm^−1^ are due to the primary amine NH_2_ stretches (Khatoon et al. 2019; Fig. 4e). Absorption peaks at 1764 cm^−1^ and 1668 cm^−1^ are attributed to the stretching frequencies of carbonyl of β-lactam (C = O) and carbonyl of amide (C = O), respectively (Khatoon et al. 2019). Absorption peaks appearing at 1457 cm^−1^ and 1164 cm^−1^ corresponds to the stretching frequencies of C = C and C–O bonds (Khatoon et al. 2019). FTIR spectrum for BCD-ZnO NPs, exhibited peak in the region of 3500–3200 cm^−1^, which corresponds to the O–H group. Suppressed peak at 1747 cm^−1^ corresponding to the stretching frequency of carbonyl of β-lactam (C = O) was observed. Merging of peaks occurred in the region of 1668–1500 cm^−1^ which is due to the carbonyl of amide (C = O) of ampicillin and H–O–H bond of beta-cyclodextrin. Peak shifting was observed at 1454 cm^−1^ and 1132 cm^−1^ which corresponds to the stretching frequencies of C = C and C–O bonds. Broad peak appeared in the region of 550–400 cm^−1^ which is due to the stretching vibration of Zn–O bond. The increase in peak intensity and shifting of peaks indicates the interaction of ampicillin with ZnO-BCD NPs (Fig. 4e).

Amphotericin B FTIR spectrum showed peak in the region of 3500–3200 cm^−1^ due to the –OH groups (Gagoś andArczewska 2010; Fig. 4f). Peaks at 2939 cm^−1^ and 2868 cm^−1^ are the C–H stretching frequencies. Peaks at 1722 cm^−1^ and 1694 cm^−1^ shows stretching vibrational frequencies of carbonyl (C = O) of ester and carboxylic acid. Sharp peak at 1561 cm^−1^, 1172 cm^−1^ and 1070 cm^−1^ corresponds to the stretching vibrations of C = C, C–O–C and C–O bonds of amphotericin B, respectively (Gagoś andArczewska 2010). Peak in the region of 3500–3200 cm^−1^ for AMP-ZnO-BCD NPs shows –OH group while peaks at 2927 cm^−1^ and 2856 cm^−1^ assigned to the C–H stretching of amphotericin B (Kaur et al. 2019). Suppression of peaks was observed at 1718 cm^−1^ and 1690 cm^−1^ which indicated the interaction of carbonyl (C = O) of ester and carbonyl (C = O) of carboxylic acid. Absorption peak at 1640 cm^−1^ corresponds to the H–O–H bond of beta-cyclodextrin. Peaks at 1521 cm^−1^, 1182 cm^−1^ and 1037 cm^−1^ are due to the stretching vibrations of C = C, C–O–C and C–O bonds of amphotericin B, respectively (Fig. 4f). Appearance of peak in region 550–400 cm^−1^ confirm Zn–O presence.

### AFM analysis

**Fig. 5:**
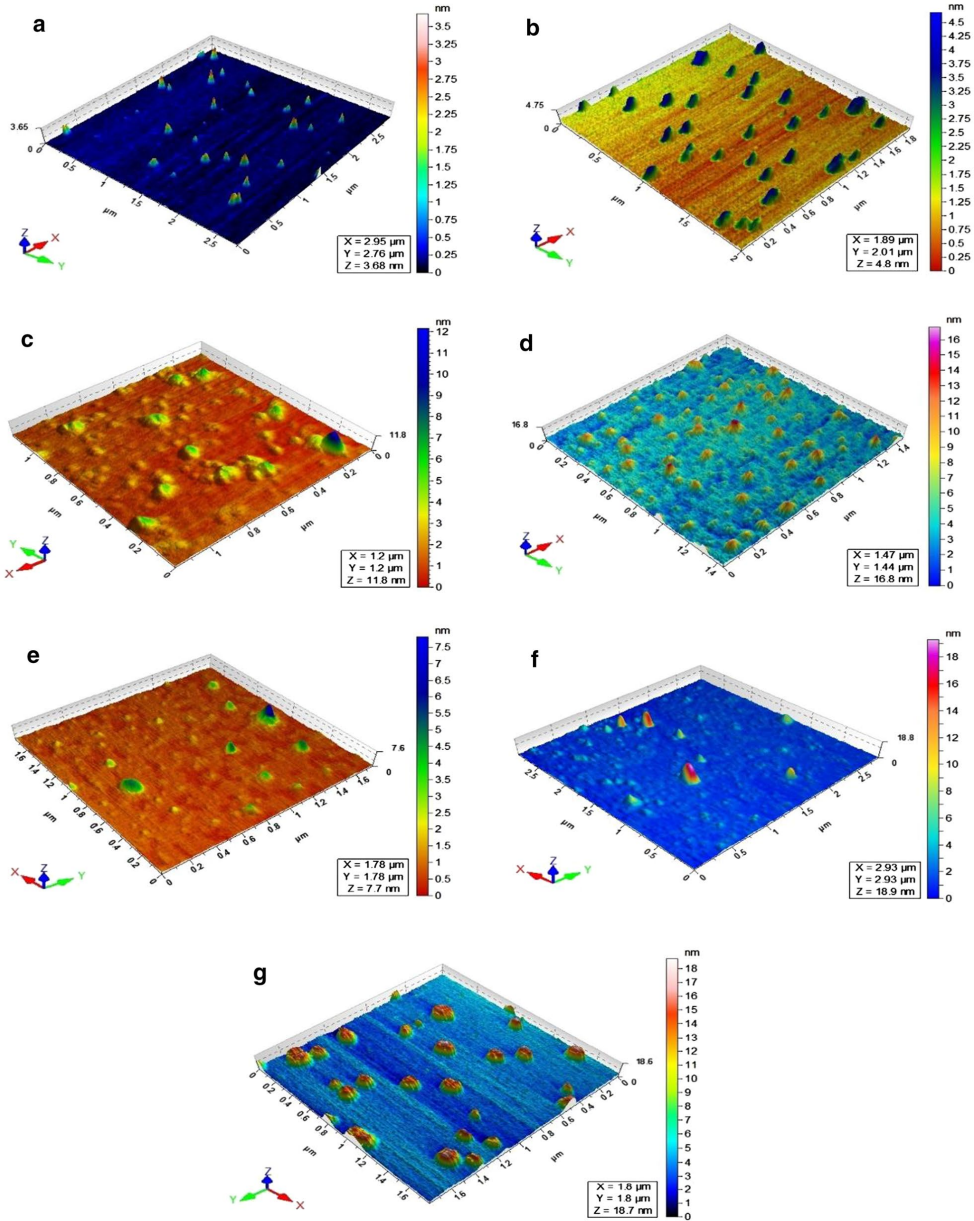
Atomic force microscopic analysis of ZnO NPs and ZnO-BCD-NPs and drugs loaded ZnO-BCD-NPs. The AFM analysis showed that ZnO-NPs alone are pointed shaped (**a**) when conjugated with BCD, it becomes bigger in size (**b**) while after drug loading over the NPs surface, the shape as well as height of nanoconjugates was significantly increased showing the successful formation of drug-NPs conjugates (**c–g**)

For the evaluation of morphological changes over the surface of ZnO-BCD NPs after loading of five drugs (Quercetin, naringin, ceftriaxone, ampicillin and amphotericin B), atomic force microscopy was performed and the results are shown in Fig. 5a–g. The AFM image of bare ZnO nanoparticles exhibited pointed shaped nanoparticles (Kalaiselvi et al. 2016) with uniform distribution over mica surface having maximum height at 3.5 nm (Fig. 5a). After the coating of BCD, the particles became broader and maximum height increases to 4.5 nm (Fig. 5b). After drugs loading, shape of the nanoparticles changed significantly. QT-ZnO-BCD NPs exhibited spherical shaped nanoparticles, which are scattered over mica surface having height around 12.0 nm (Fig. 5c). NAR-ZnO-BCD NPs showed very ordered pointed shaped nanoparticles having 16.5 nm height (Fig. 5d). CFT-ZnO-BCD NPs and AMP-ZnO-BCD NPs showed broad dome shaped morphologies having maximum height around 7.5 and 19.0 nm, respectively (Fig. 5e, f). AMB-ZnO-BCD NPs appeared as circular shaped nanoparticles having maximum height around 18.7 nm (Fig. 5g).

### ZnO-CD-drugs nanocomposite showed significant antibacterial activities against MDR pathogenic bacteria

**Fig. 6:**
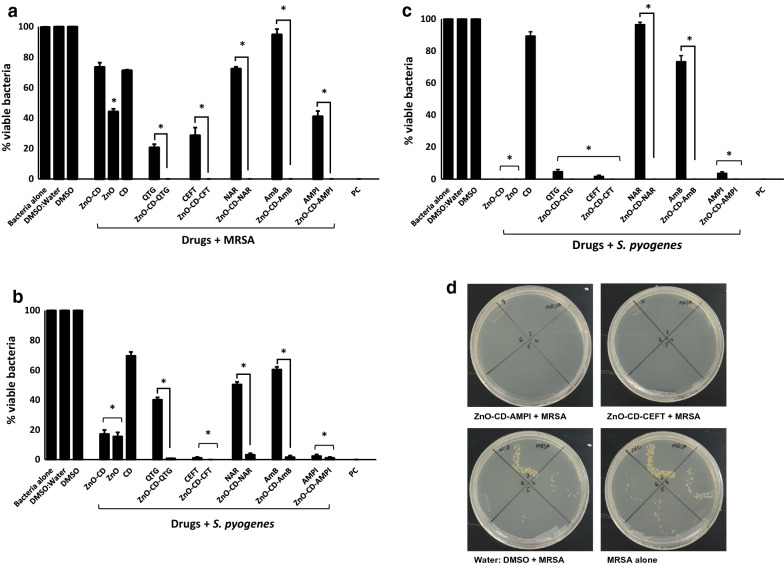
ZnO-NPs loaded with various drugs showed important antibacterial activity against Gram-positive pathogenic bacteria. Briefly, bacteria (1  ×  10^6^) were incubated with different drugs conjugated with ZnO-NPs and nanoparticles alone for two hours at 37 °C as defined in Materials and Methods. Next, the cultures were ten-fold serially diluted and plated onto the nutrient agar plates. The plates were incubated for 24 h at 37 °C and enumerated the viable bacterial colonies on the next day. The results showed that drugs conjugated ZnO-NPs exhibited significant antibacterial activity against the Gram-positive bacteria. For negative control, bacteria were incubated in PBS alone whereas for positive control gentamicin (100 µg/mL) was used. The data are representative of several experiments performed in duplicate and expressed as the mean  ±  standard error, where (*) represent when P  ≤  0.05

ZnO-NPs, drugs alone (Quercetin, Ceftriaxone, Naringin, Amphotericin B and Ampicillin), and the combination of ZnO-CD-drug were tested for their antibacterial activities against a panel of Gram-negative and Gram-positive pathogenic bacteria. The results revealed that drugs and drug conjugated ZnO-NPs showed significant bactericidal activities against MRSA (P  ≤  0.05; Fig. 6a). ZnO-CD complex and cyclodextrin, NAR and AMB did not show antibacterial properties whereas, rest of the drugs and their NPs conjugates showed significant bactericidal effects against MRSA. Drug alone and drug-NPs comparison showed that the NPs further enhance their bactericidal properties against MRSA. When tested against *S. pyogenes*, all the drugs and their NPs counterparts except CD exhibited antibacterial effects (Fig. 6b). When drug alone and their NPs was compared, there is no significant differences between the drug and their NPs for ceftriaxone and ampicillin. All drugs and their NPs except CD and NAR showed extraordinary antibacterial activities against *S. pneumoniae* (Fig. 6c).

**Fig. 7:**
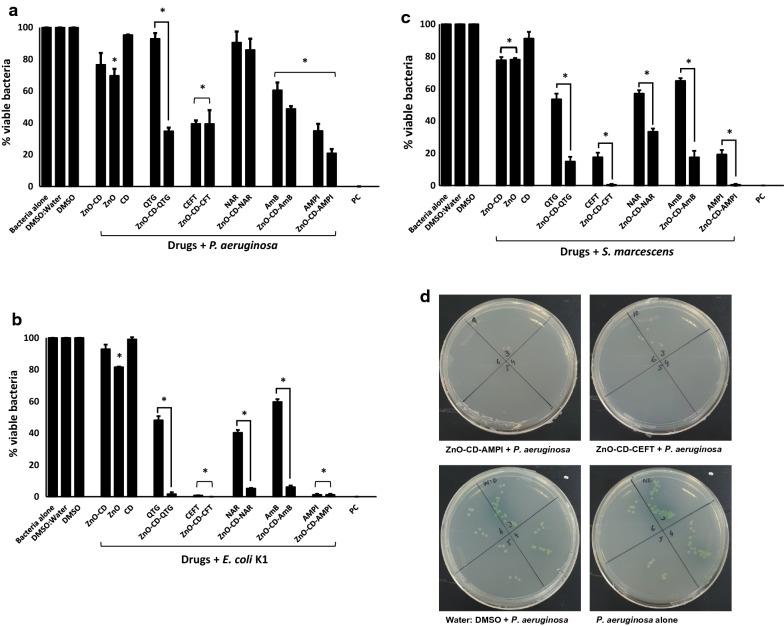
Treatment with ZnO-NPs loaded with various drugs eliminated Gram-negative pathogenic bacteria. Briefly, ZnO-NPs and drug conjugates were mixed with test bacteria for 120 min at 37 °C. After this incubation, cultures were serially diluted and plated on nutrient agar plates. The plates were incubated at 37 °C for 24 h and subsequently viable bacteria were counted upon this incubation. For controls, bacteria incubated alone in PBS and with gentamicin (100 µg/mL) was used as negative and positive controls, respectively. ZnO-NPs conjugated drugs showed remarkable antibacterial properties against Gram-negative bacteria. The data are representative of several experiments performed in duplicate and expressed as the mean  ±  standard error, where (*) represent when P  ≤  0.05

Among Gram-negative bacteria, all drugs except ZnO-CD, CD, QTG, NAR and its NPs, showed bactericidal activities against *P. aeruginosa* (P  ≤  0.05; Fig. 7a). More importantly, among all the drug-NPs conjugate ZnO-CD-QTG showed bactericidal activities compared to its drug i.e., QTG. When tested against *E. coli* K1, all the drugs and their NPs except ZnO-CD and CD alone presented bactericidal activities (Fig. 7b). In case of drug-NPs comparison, all the NPs augments the antibacterial activity except ampicillin. For *S. marcescens,* all the drug except CD revealed antibacterial properties. Moreover, the NPs exceptionally increases the antibacterial potency of the drugs (Fig. 7c). The MIC_50_ values of ZnO-NPs and drug encapsulated ZnO-NPs are summarized in Table 4. The overall results showed that ZnO and drug loaded NPs presented substantial antibacterial activity against the clinical isolates.

**Table 4:** MIC_50_ (μg/mL) of ZnO-NPs and drugs-loaded ZnO-NPs suspension against Gram-negative and Gram-positive bacteria

Bacteria	CEFT	ZnO-CEFT	QTG	ZnO-QTG	NAR	ZnO-NAR	AMB	ZnO-AMB	AMPI	ZnO-AMPI	ZnONPs
	MIC_50_	MIC_50_	MIC_50_	MIC_50_	MIC_50_	MIC_50_	MIC_50_	MIC_50_	MIC_50_	MIC_50_	MIC_50_
*E. coli* K1	23	14	> 500	> 500	> 500	> 500	> 500	> 500	123	64	290
*P. aeruginosa*	130	128	> 500	340	> 500	> 500	> 500	390	110	95	255
*S. marcescens*	55	19	310	245	350	275	470	325	56	21	245
MRSA	16	4	400	114	> 500	270	> 500	236	170	118	158
*S. pyogenes*	14	6	340	125	420	189	485	210	26	18	130
*S. pneumoniae*	11	4	90	55	88	52	> 500	270	22	9	95

### ZnO-CD drug conjugates exhibited minimal cytotoxicity

**Fig. 8:**
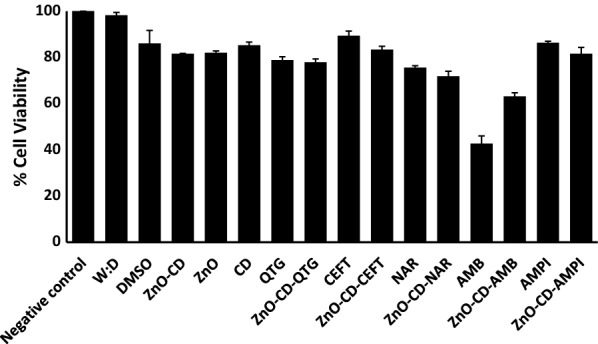
Treatment with ZnO-NPs and drug conjugates exhibited minimal cytotoxicity against human cell lines. Human cells were grown in 96 well plate up to 80–90% confluency as discussed in material and methods. Next, cells monolayer was challenged with drugs-NPs combinations for 24 h at 37 °C in humidified conditions with 5% CO_2_. Cells alone in RPMI was taken as negative control. The data are representative of several experiments performed in duplicate and expressed as the mean  ±  standard error, where (*) represent when P  ≤  0.05. Data was analyzed using Graph Pad Prism software (8.0.2)

Cytotoxic effects of drugs/drug-NPs on human cell lines were determined using MTT assays. The results showed that the majority of drugs/drug-NPs showed minimal cytotoxicity upon overnight incubation (Fig. 8). Amphotericin B and its NPs conjugates counterpart showed 57% and 37% (moderate to high) cytotoxic effects against human cells respectively.

## Discussion

Nano-biotechnology is receiving considerable attention to design new antibacterials (Seil and Webster 2012). Silver NPs (AgNPs) are well known to exert broad spectrum antibaterial effects (Guzman et al. 2012; Tamboli and Lee 2013). Nanocomposite of AgNPs and reduced graphine oxide (rGO) displayed antibatecial activity against a range of bacteria including *Proteus mirabilis, E. coli* and *S. aureus* (Habash et al. 2014, 2017; Prasad et al. 2017). In additon to AgNPs, copper oxide (CuO) and ZnO NPs have shown promising antibacterial activities (Azam et al. 2012). Further, to enhance the efficacy of antibacterials, here we have synthesized ZnO-NPs, loaded with β-cyclodextrin and five drugs (Quericitin, Ceftrixon, Naranjin, Amphotericin B and Ampicillin). These formulations were characterized using a range of techniques such as zeta potential analysis and zeta sizer, UV-visible, FTIR, AFM as previously described (Gagoś and Arczewska 2010; Ethiraj et al. 2014; Catauro et al. 2015; Liu et al. 2015; Diaz-Uribe et al. 2016; Kalaiselvi et al. 2016; Kavitha et al. 2017; Mushtaq et al. 2017; Reddy et al. 2020; Kadam et al. 2020; Kaur et al. 2020; Yang et al. 2020; Sathishkumar et al. 2021). ZnO-NPs were synthesized using direct precipitation method and upon successful formation coupled with β-cycldextrin. Here for first time, we have conjugated ZnO-NPs with these drugs and evaluated for their antibacterial properties against a panel of Gram-positive and Gram-negative bacteria. ZnO-NPs exhibited broad spectrum antibacterial activities against clinical isolates tested in this study which is supported by prvious study (Jones et al. 2008). *E. coli* showed resistance towards ampicillin and cephalosporins (Jacoby 2009; Uzunović-Kamberović et al. 2010; Mendoza-Palomar et al. 2017). *E. coli* also showed resistance to naringenin (metabolite of naringin) rich compounds (Agus et al. 2017). *S. marcescens* are thought to show resistance against all cephalosporin including ceftriaxone (Sleigh 1983). Similarly, *P. aeruginosa* exhibited resistance against ceftriaxone (cephalosporin) and ampicillin (Lamb et al. 2002; Rafailidis et al. 2007; Khatoon et al. 2019). MRSA harbour *mecA* gene which is key factor in resistance against ampicillin (Rafailidis et al. 2007). However, by conjugating these drugs with ZnO NPs, the antibacterial effects were significantly enhanced against tested bacteria. ZnO-NPs possess large surface area with small size that enable NPs to exhibit profound antibacterial activity. It is also believed that NPs trigger the reactive oxygen species (ROS), thus affecting bacterial cell integrity (Sirelkhatim et al. 2015). ZnO-NPs and their nanocomposites with drugs were evaluted for their MIC_50_ against a range of Gram-positive and Gram-negative bacteria. Souza et al. (2019) also identified MIC values of ZnO-NPs and nanoconjugates against food-borne pathogenic bacteria and found them effective (Souza et al. 2019). Similarly, Emami-Karvani and Chehrazi (2011) determined the MIC of ZnO-NPs against *E. coli* and *S. aureus*. The antibacterial effects were higher against *S. aureus* and lower against *E. coli* (Emami-Karvani and Chehrazi 2011).

Notably, two antibiotics, Ceftriaxone and Ampicillin are presently used to treat various types of bacterial infections. Ceftriaxone is a broad-spectrum cephalosporin antibiotic used currently for the treatment of bacterial infections in various locations, such as in the respiratory tract, skin, soft tissue, and urinary tract. The mode of action involves inhibition of bacterial cell wall synthesis. Similarly, Ampicillin is used currently to treat various types of bacterial infections including respiratory and urinary tracts. It inhibits cell wall synthesis by targeting transpeptidase, leading to cell lysis. Our findings are promising, in that, the efficacy of both antibiotics can be enhanced by conjugation with ZnO NPs. As the pharmacokinetic and pharmacodynamic profiles of both antibiotics are well established, it is logical to test these compounds and determine their translational value and it is the subject of future studies. In addition, future research will determine whether the aforementioned conjugated antibiotics can overcome increasingly resistant strains of bacteria.

Notably, ZnO-NPs and drug conjugated ZnO-NPs showed minimal cytotoxic effects against human cell lines. Our results are in the agreement with previously published data. For example, Colon et al. (2006) reported that ZnO-NPs exhibited negligible cytotoxic activities against human cell lines (Colon et al. 2006). Similarly, El-Waseif (2019) reported ZnO NPs as non-toxic against vero cell lines below 300 µg/mL (El-Waseif 2019). In other studies, ZnO-NPs showed significant anticancer activity against human adinocarcinnoma (MCF-7) (Prashanth et al. 2015; Anitha et al. 2018). Taken together, the present study revealed that ZnO-NPs and drugs-loaded ZnO-NPs exhibited promising antibacterial activities against the MDR Gram-positive and Gram-negative bacteria. The NPs and their drugs conjugates showed concetration-dependant bactericidal activities. Finally, NPs showed negligible cytotoxicty against human cell line suggesting their potenial applications as effective chemotherapeutic candidates against infections caused by MDR bacteria.

## Supplementary Information

**Additional file 1: Figure S1.** Chemical structure of drugs (**a**) quercetin (QT), (**b**) naringin (NAR), (**c**) ceftriaxone (CFT), (**d**) ampicillin (AMP) and (**e**) amphotericin B (AMB). **Figure S2.** Calibration curve of quercetin (QT). **Figure S3.** Calibration curve of naringin (NAR). **Figure S4.** Calibration curve of ceftriaxone (CFT). **Figure S5.** Calibration curve of ampicillin (AMP). **Figure S6.** Calibration curve of amphotericin B (AMB).

## Data Availability

All relevant data and material is given in the manuscript.
