# Supplementary material for: Zinc oxide nanoparticles conjugated with clinically-approved medicines as potential antibacterial molecules
Source: AMB Express. 2021 Jul 10;11:104. doi: 10.1186/s13568-021-01261-1 (PMC8272778; doi:10.1186/s13568-021-01261-1)

AMB Express

Manuscript title: Zinc oxide nanoparticles conjugated with clinically-approved medicines as potential antibacterial molecules

[1]Noor Akbar†, [2]Zara Aslam†, [1]Ruqaiyyah Siddiqui, [2]Muhammad Raza Shah, [3*]Naveed Ahmed Khan.

[1]College of Arts and Sciences, American University of Sharjah, University City, Sharjah 26666, United Arab Emirates; [2]H.E.J. Research Institute of Chemistry, International Centre for Chemical and Biological Sciences, University of Karachi, Karachi, 75270, Pakistan; [3]Department of Clinical Sciences, College of Medicine, University of Sharjah, University City, Sharjah, 27272, Unites Arab Emirates.

† Both authors contributed equally.

Running title: ZnO drugs and antibacterials

*Corresponding address: N. A. Khan, Department of Clinical Sciences, College of Medicine, University of Sharjah, University City, Sharjah, 27272, Unites Arab Emirates.

Tel: +971-6505-7722. Fax. +971-6515-7244. E-mail: naveed5438@gmail.com

Figure S1. Chemical structure of drugs **(a)** quercetin (QT), **(b)** naringin (NAR), **(c)** ceftriaxone (CFT), **(d)** ampicillin (AMP) and **(e)** amphotericin B (AMB)

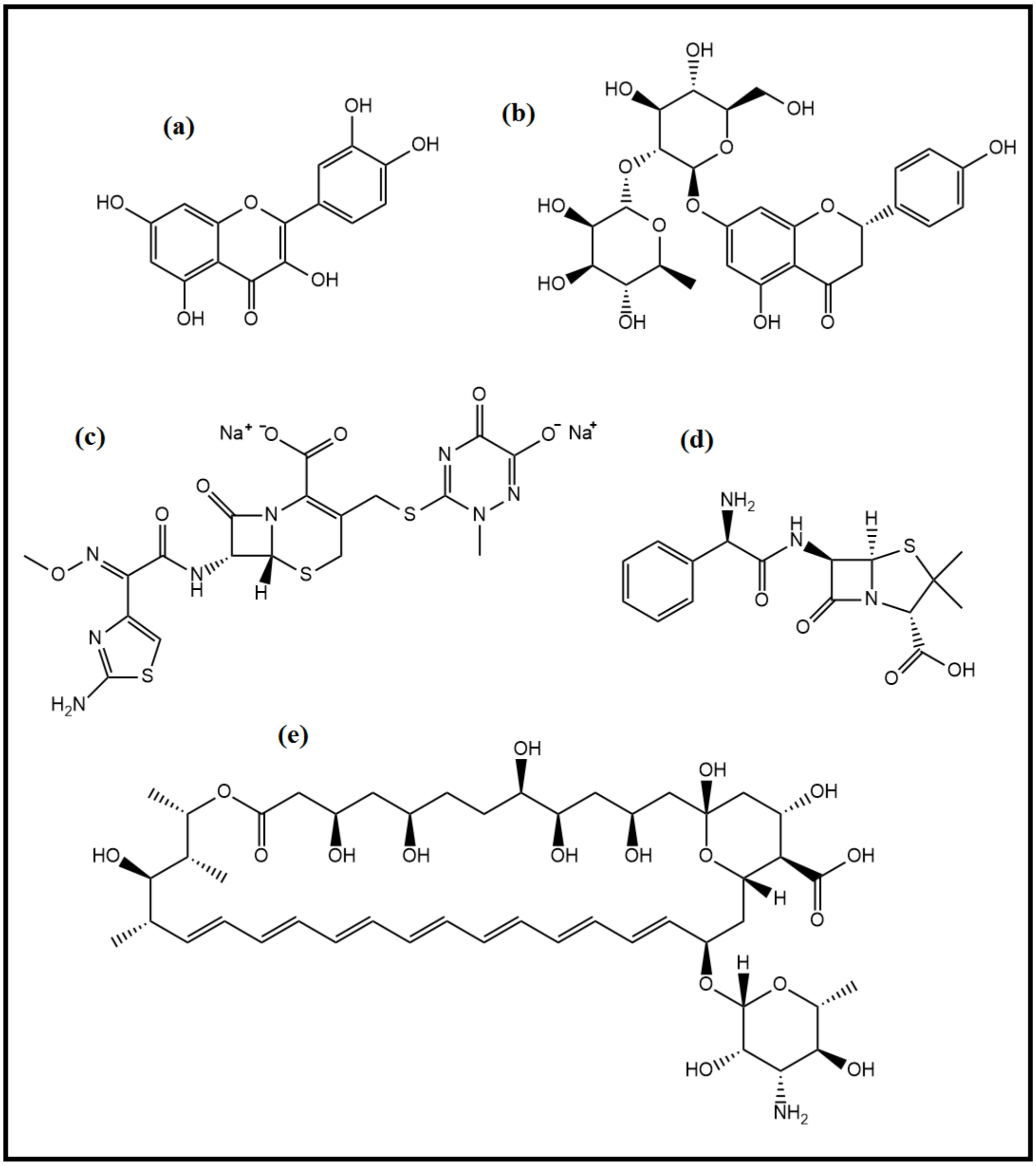

Figure S2. Calibration curve of quercetin (QT)

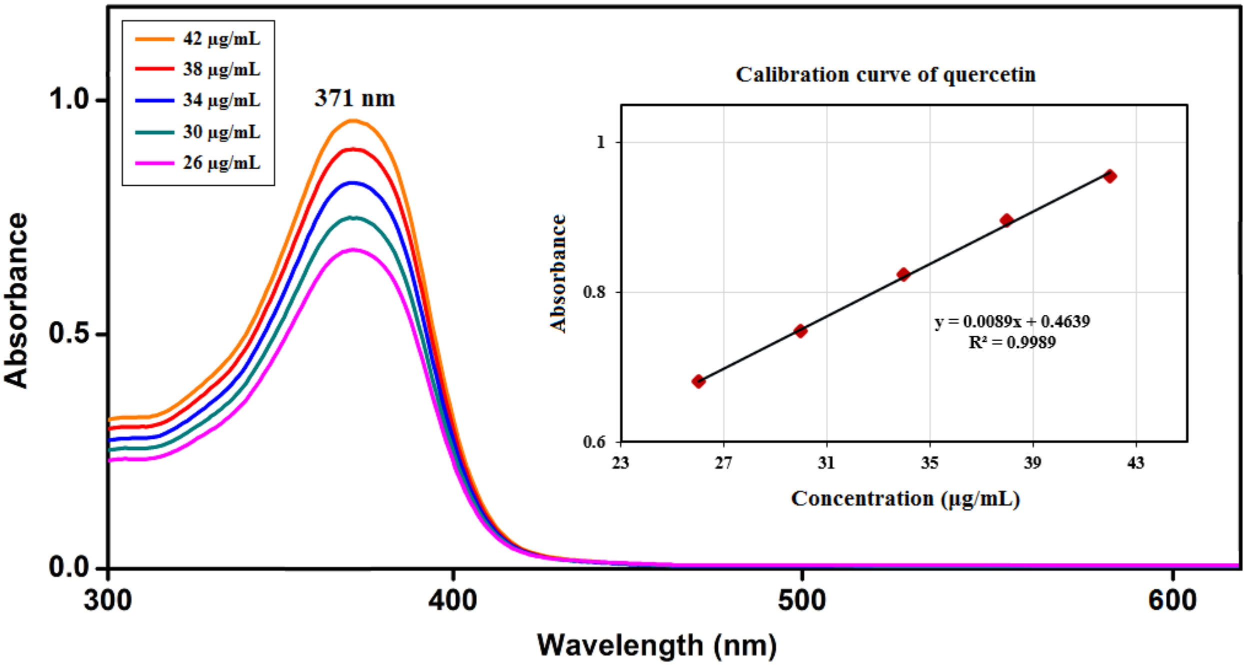

Figure S3. Calibration curve of naringin (NAR)

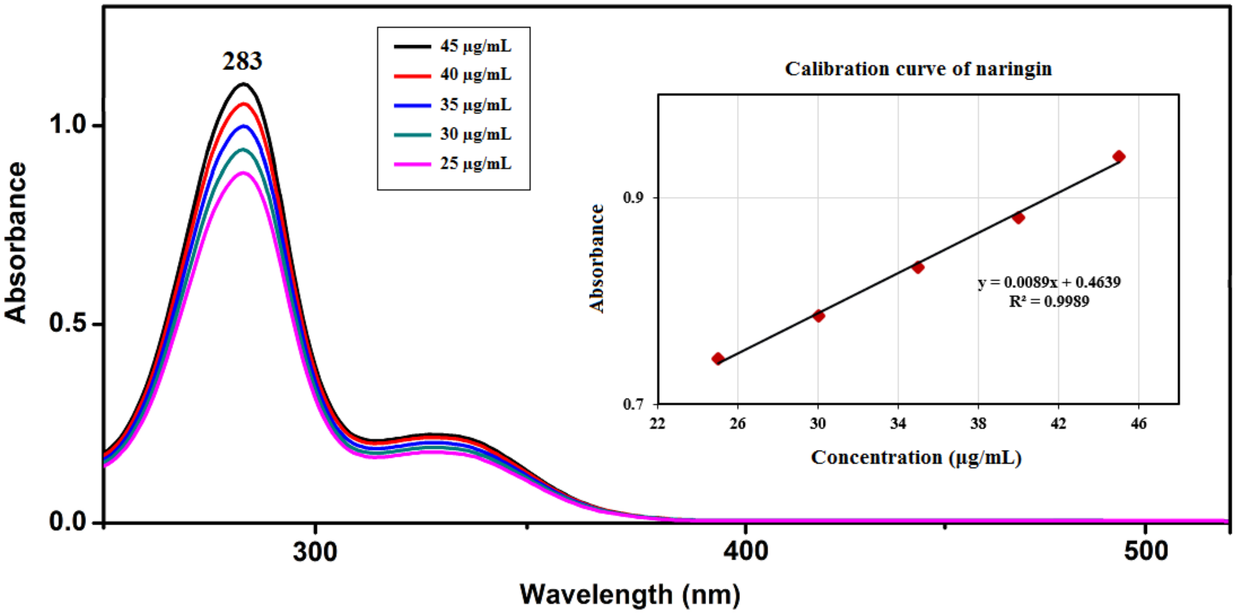

Figure S4. Calibration curve of ceftriaxone (CFT)

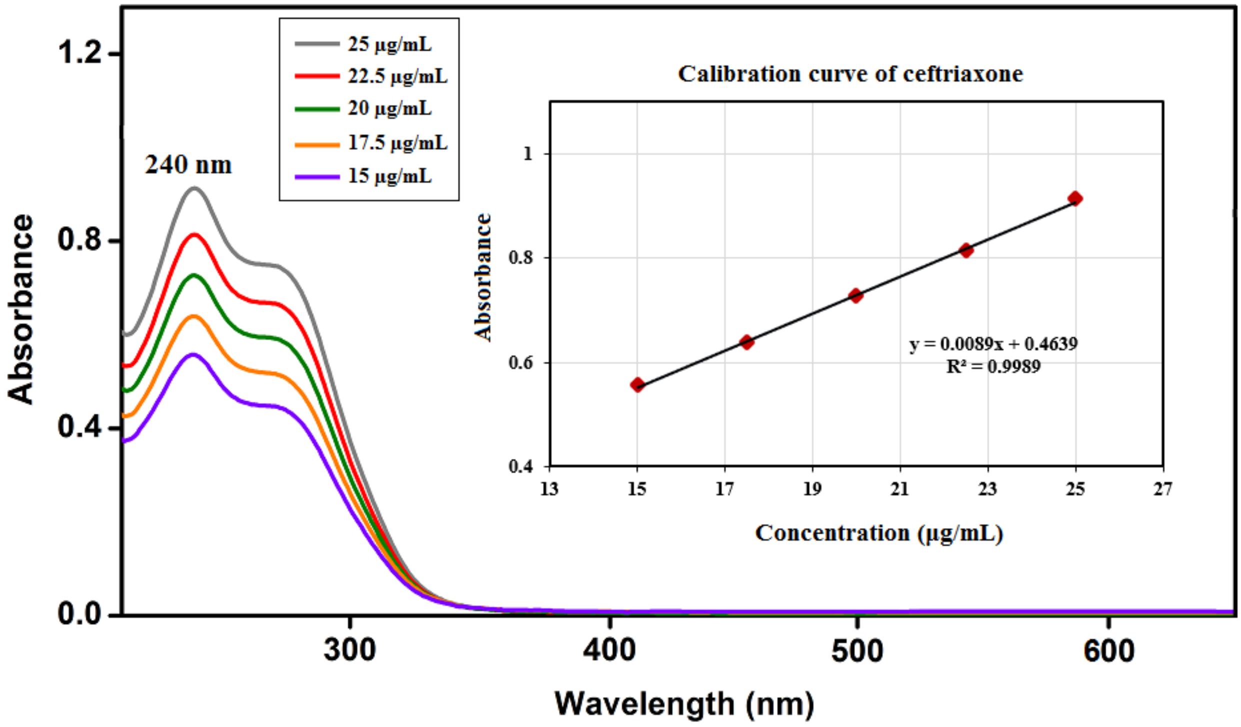

Figure S5. Calibration curve of ampicillin (AMP)

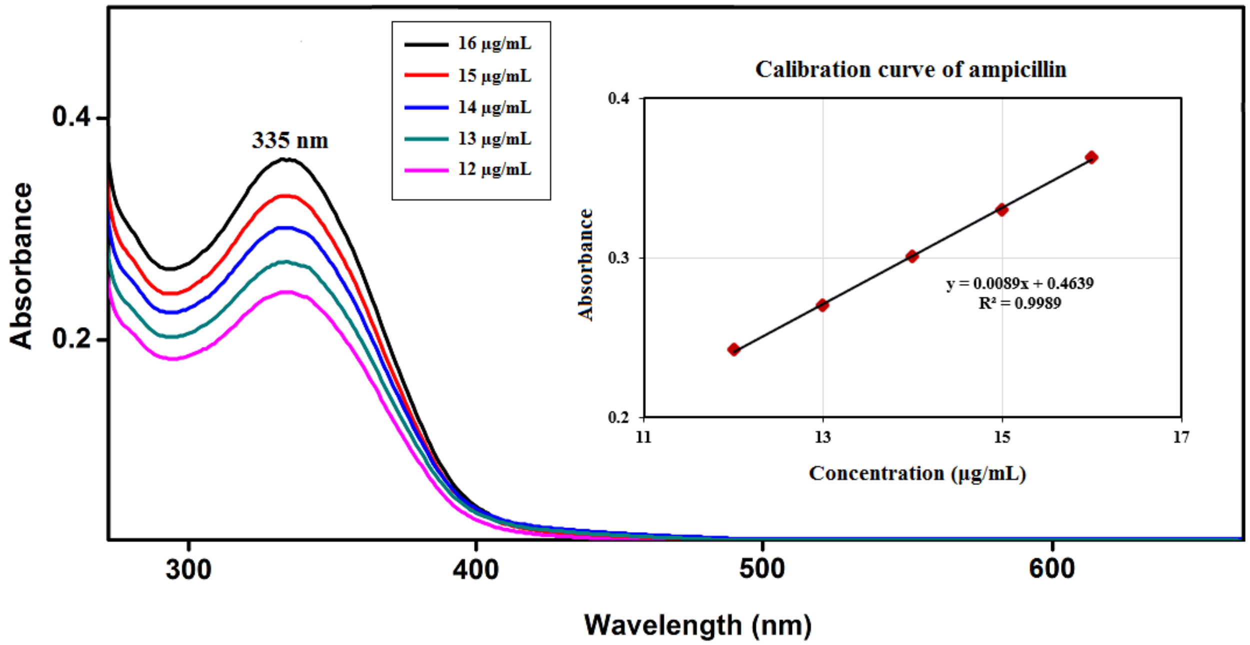

Figure S6. Calibration curve of amphotericin B (AMB)

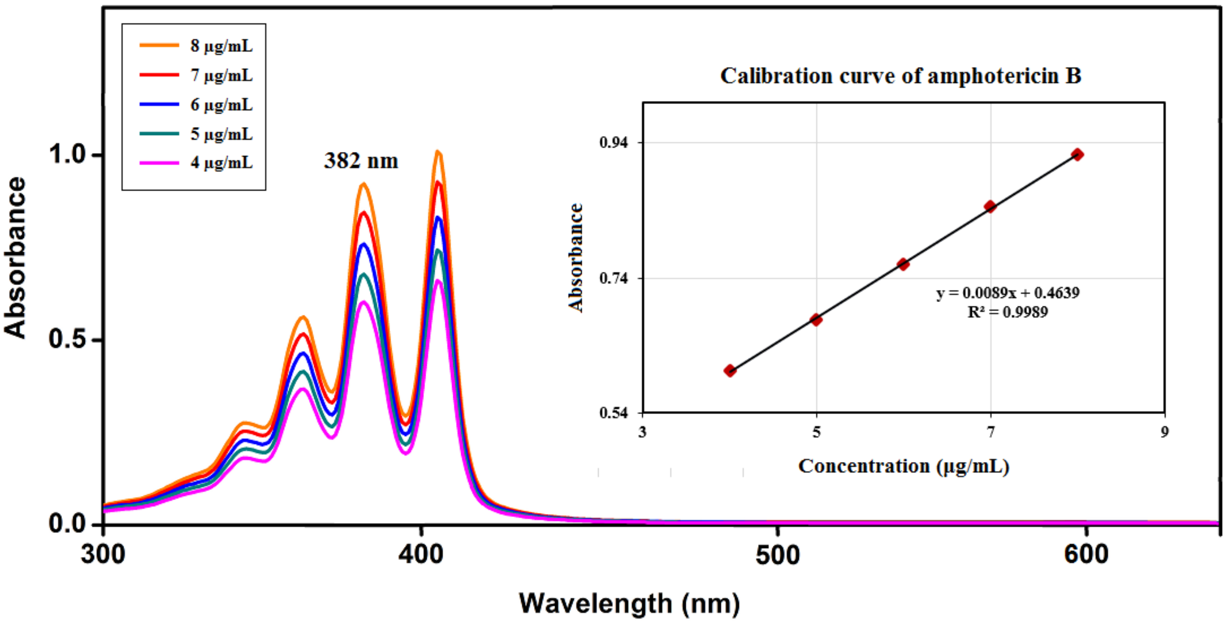

Supplement: Supplementary file 1 — Additional file 1: Figure S1. Chemical structure of drugs (a) quercetin (QT), (b) naringin (NAR), (c) ceftriaxone (CFT), (d) ampicillin (AMP) and (e) amphotericin B (AMB). Figure S2. Calibration curve of quercetin (QT). Figure S3. Calibration curve of naringin (NAR). Figure S4. Calibration curve of ceftriaxone (CFT). Figure S5. Calibration curve of ampicillin (AMP). Figure S6. Calibration curve of amphotericin B (AMB). [file 13568_2021_1261_MOESM1_ESM.pdf]
